# Commentary: Integrated blockchain-deep learning approach for analyzing the electronic health records recommender system

**DOI:** 10.3389/fpubh.2023.1133142

**Published:** 2023-04-05

**Authors:** Siwan Noh, Muhammad Firdaus, Kyung-Hyune Rhee

**Affiliations:** ^1^Department of Information Security, Pukyong National University, Busan, Republic of Korea; ^2^Department of Artificial Intelligence Convergence, Pukyong National University, Busan, Republic of Korea; ^3^Division of Computer Engineering, Pukyong National University, Busan, Republic of Korea

**Keywords:** electronic health record (EHR), blockchain (BC), information security, access control, inter planetary file system (IPFS)

## 1. Introduction

Mantey et al. (1) proposed an integrated blockchain-deep learning environment for analyzing electronic health records (EHRs). They highlighted the limitations of traditional EHR management systems and argued that their system could overcome these shortcomings. However, the details of their system were not provided in the paper, so we referred to their previously published research papers (2, 3) to analyze and gain insights into our findings. In these papers, they proposed a recommendation system that leverages blockchain and deep learning in various scenarios. Although they presented different scenarios and recommendation systems, the approach for storing and sharing EHRs on the blockchain remained unchanged. We conducted a security analysis of this approach and identified some security vulnerabilities that need to be addressed. Their method of controlling access to EHR was inefficient, burdening patients with complicated and wasteful activities due to improper access control methods. Although they aimed to eliminate dependency on a centralized system, their algorithm failed to ensure confidentiality for the system administrator. In an attempt to safeguard the content identifier, they applied an additional encryption procedure, which was impractical and unnecessary for both public and private IPFS networks. Consequently, it is challenging to evaluate whether their proposed system meets users' requirements effectively.

## 2. Discussion

In Mantey et al. (1), the authours used a private IPFS network (4) and a permissioned blockchain in their system. To summarize their approach, EHR is stored in the decentralized P2P storage, and patients control access to their EHR through immutable blockchain. They propose an algorithm to guarantee the confidentiality of EHRs stored in the IPFS network and blockchain; however, they did not provide a detailed explanation of the algorithm in Mantey et al. (1). Thus, we analyze their previous works (2, 3) and reconstruct the detailed description of the algorithm based on these papers as shown in Algorithm 1. Table 1 shows notations used in this algorithm.

**Algorithm 1:** Creating and updating health records in Hyperledger blockchain.

**Function 1. Retrieve and Re-encrypt EHR by the Administrator:**
Input: $msk,cid_{C_{0}}$
Output: κ_*u*_, *C*_2_, *cid*_*C*_1__
1. If requester's ID ∈ patient *p*'s ACL, the administrator retrieves *p*'s EHR $D_{i}$ from IPFS
1) $C_{0}$ ← $retrieveData\left(cid_{C_{0}}\right)$
2) $D_{i}$ ← $decryptS\left(C_{0},msk\right)$
2. Generate session key *sk*
3. Re-encrypt D under the new symmetric key *sk*
1) $C_{1}$ ← encryptS($D_{i}$, *sk*)
2) κ_*u*_ ← *encryptA*(*sk, puk*_*u*_)
3) $cid_{C_{1}}\leftarrow uploadData\left(C_{1}\right)$
4) $C_{2}$ ← $encryptA(cid_{C_{1}}$, *puk*_*u*_)
4. Administrator send κ and $C_{2}$ to authorized users (e.g., patient and doctor)
**Funtion 2. Update EHR by authorized users:**
Input: $\kappa_{u},C_{2}$
Output: $cid_{D_{i+1}}$
1. Decrypt *C*_2_ and retrieve *C*_1_ from IPFS network
1) $cid_{C_{1}}$ ← $decryptA(C_{2}$, *prk*_*u*_)
2) $C_{1}$ ← $retrieveData\left(cid_{C_{1}}\right)$
2. Decrypt session key and get EHR $D_{i}$
1) *sk*←d*ecryptA*(κ_*u*_, *prk*_*u*_)
2) $D_{i}$ ← $decryptS\left(C_{1},sk\right)$
3. Update EHR and upload new EHR
1) $D_{i+1}$ ← $D_{i}+D_{update}$
2) $C_{0}^{{}^{\prime }}\leftarrow encryptS\left(D_{i+1},msk\right)$
3) $cid_{C_{0}^{{}^{\prime }}}$ ← $uploadData\left(C_{0}^{{}^{\prime }}\right)$

**Table 1:** Notations and descriptions.

**Notation**	**Description**
*msk*	The master secret key of the system administrator
*sk*	symmetric key
*prk*_*u*_, *puk*_*u*_	asymmetric key pair for the user *u*
*cid*_*target*_	The content identifier to retrieve *target* from IPFS network
*encryptS*(*m, k*)	symmetric encryption under the key *k* for the message *m*
d*ecryptS*(*m, k*)	symmetric decryption under the key *k* for the message *m*
encryptA(*m, k*)	asymmetric encryption under the key *k* for the message *m*
d*ecryptA*(*m, k*)	asymmetric decryption under the key *k* for the message *m*
*retrieveData*(*cid*)	retrieve data linked to *cid* from IPFS network
*uploadData*(*d*)	upload the data *d* to IPFS network and record its *cid* on the blockchain.

Based on the Algorithm 1, we can outline that the main objective of their approach is to ensure that only the authorized requester can access the EHR. They claimed their approach could achieve these objectives by sharing encrypted EHR under new encryption keys for authorized users and storing the Access Control List (ACL) on the immutable blockchain. However, we remark on some potential security vulnerabilities to be further considered. In the rest of the paper, we provide our analysis and insight into Mantey et al.'s research.

### 2.1. Improper access control

The authors' system stores a list of EHR and access rights in a blockchain. According to Algorithm 1, permission granted to a requester (clinicians, doctors, and healthcare providers) is validated based on the ACL before data sharing. However, the ACL is not proper for EHRs sharing scenarios discussed in the research, according to National Institute of Standards and Technology Interagency Report 7316 (5), which deals with the assessment of access control systems. As the basis for our argument, we present the limitations of the ACL in the large system mentioned in the report. The report highlights the limitations of the ACL in large systems, as it becomes challenging to determine all privileges for a user, not just for a specific object. For instance, to retrieve all access rights granted to someone, one must examine all ACLs in the system, which can be difficult as the system grows larger. Moreover, if a data requester leaves the system, all patients in the system will have to search their ACLs to revoke the access rights granted to him. The management of ACLs becomes increasingly challenging as the system grows larger.

### 2.2. Privileged access

The authors' major contribution is addressing trust issues in centralized storage. To mitigate this, they implemented a decentralized storage environment to store encrypted EHR. They designed the system to only allow authorized users to access it, and used transport encryption (6) to ensure the confidentiality of the EHR on the network. Transport encryption is a technique that encrypts data between two parties who share data under a shared secret key to ensure data confidentiality to third parties. A key agreement or key transport scheme is used to securely establish a shared secret key between the two parties (7). However, they stated that the system administrator responsible for managing EHR generates and transports this shared secret key. This means that the administrator can access all EHRs in the system at any time without the patient's permission. As seen in Algorithm 1, the administrator's master secret key *msk* encrypts the patient's EHR $D_{i}$. If the request is valid, the encrypted EHR $C_{0}$ is re-encrypted under a new symmetric key *sk*. The re-encrypted EHR $C_{1}$ is then shared with requesters through the IPFS network. Thus, despite their claim to have solved the issues with traditional central storage environments, EHR is still under complete control of the central organization.

### 2.3. Redundant procedure

Traditional centralized web protocols, such as HTTP, use location-based addressing to locate where data is stored. In contrast, IPFS uses content-based addressing, which assigns a unique content identifier (CID) derived from the data itself. In Mantey et al. (1), the authors upload re-encrypted ciphertext $C_{1}$ to the IPFS network and obtain its CID $cid_{C_{1}}$. They then encrypt *sk*, $cid_{C_{1}}$ under the requester's public key *puk*_*u*_ and send the resulting encrypted CID $C_{2}$ and secret key κ_*u*_ to the requester to share the requested EHR. However, not disclosing the CID explicitly does not guarantee that stored data will not be found by third parties. All upload and retrieve requests in the IPFS network are announced to connected nodes until the request reaches the destination node, enabling any IPFS node to monitor the network and retrieve data without knowing the CID.^1^ Therefore, whether or not the CID is encrypted, a third party can retrieve the EHR stored in IPFS, and even if the EHR is retrieved, it is secure because it is encrypted. As a result, the authors' approach of encrypting the CID under the requester's public key has no security implications.

## 3. Conclusion

Our main argument is summarized as follows.

**Improper access control**: The use of the ACL may not be suitable for large, dynamic systems such as the one proposed by the authors. Therefore, the authors should consider alternative access control models that are appropriate this environment. One alternative to consider is a role-based access control (RBAC) model, which is more efficient for large systems because it assigns permissions to specific small groups. In the RBAC model, patients do not need to maintain access control policies because only requesters with specific roles defined by patients can access EHR. The authors should consider using the RBAC model instead of an ACL that explicitly records access rights. By doing so, they can ensure that access to EHR is restricted to only those with specific roles authorized by the patient, without requiring the patient to maintain complex access control policies.

**Privileged access**: To ensure the confidentiality of patients' sensitive data and prevent unauthorized third-party access, a proper encryption mechanism must be designed. However, the authors give the system administrator unrestricted access for managing EHRs, which diminishes the benefits of integrating blockchain. Patient privacy should be guaranteed for all users in the system, apart from the authorized requester. To guarantee the confidentiality of EHRs, they may consider leveraging cryptographic techniques such as role-based encryption (RBE) or proxy re-encryption (PRE). RBE is a cryptographic technique that combines the RBAC model with encryption. It embeds RBAC access policies within encrypted data, enabling users with authorized roles to access the data by decrypting it. As an alternative, PRE allows a third party, referred to as the proxy, to transform an encrypted message from one public key to another without knowing the private keys. The proxy can permit the transfer of encrypted data among different users or systems without having access to the data itself or the ability to read or modify it. Both cryptographic techniques should prevent the central authority from accessing the encrypted data without the necessary access privileges, ensuring the privacy of patients' sensitive data. By leveraging RBE or PRE, the authors can ensure that only users with authorized roles can access the EHR data, thereby increasing the security of the system.

**Redundant procedure**: The authors propose encrypting the CID and shared secret key under the requester's public key to ensure that only authorized users can retrieve the ciphertext from IPFS. However, this approach is meaningless from a security perspective since all IPFS nodes already have access to the CID and its information. Moreover, obtaining plaintext from the ciphertext using the CID is unnecessary since it has no relation to shared secret key. Even if a third party obtains the CID, they cannot access the plaintext without the shared secret key. Furthermore, because the private IPFS network only involves authorized servers, external attackers cannot obtain the ciphertext. Thus, encrypting the CID is redundant since there are already other methods in place to achieve the same goal.

We believe that further discussion about their proposed research is required, and we hope that this will contribute to their research.

## Author contributions

All authors listed have made a substantial, direct, and intellectual contribution to the work and approved it for publication.

## Footnotes

^1^“ipfs-search” https://ipfs-search.com (accessed September 27, 2022).
